# Effect of chloride ions on persulfate/UV-C advanced oxidation of an alcohol ethoxylate (Brij 30)

**DOI:** 10.1038/s41598-024-66442-x

**Published:** 2024-07-03

**Authors:** Çisem Ecer Uzun, Işık Kabdaşlı

**Affiliations:** Environmental Engineering Department, Civil Engineering Faculty, İstanbul Technical University, Ayazağa Campus, 34469 Sarıyer, İstanbul Turkey

**Keywords:** Alcohol ethoxylate, Brij 30, Bicarbonate ions, Chloride ions, Persulfate/UV-C oxidation, Water/wastewater matrix, Environmental sciences, Energy science and technology

## Abstract

In the present study, the effect of chloride ions on the oxidative degradation of an alcohol ethoxylate (Brij 30) by persulfate (PS)/UV-C was experimentally explored using Brij 30 aqueous solution (BAS) and a domestic wastewater treatment plant effluent spiked with Brij 30. Brij 30 degradation occurred rapidly during the early stages of oxidation without affecting the water/wastewater matrix. Mineralization of intermediates of Brij 30 degradation markedly influenced by presence of chloride ions. Chloride ions at concentrations up to 50 mg/L accelerated the mineralization through reactions involving reactive chlorine species, which reduced the sink of SO_4_^·−^ by Cl^−^ scavenging at both initial pH of 6.0 and 3.0 in the case of BAS. The fastest mineralization was achieved under acidic conditions. The WWTP effluent matrix significantly influenced mineralization efficacy of the intermediates. Co-existence of ${\text{HCO}}_{3}^{-}$ and Cl^−^ anions accelerated the mineralization of degradation products. Organic matter originating from the WWTP effluent itself had an adverse effect on the mineralization rate. The positive effects of organic and inorganic components present in the WWTP effluent were ranked in the following order of increasing influence: (Organic matter originating from the effluent + Cl^−^ +${\text{HCO}}_{3}^{-}$) < (Cl^−^) < (Cl^−^ +${\text{HCO}}_{3}^{-}$).

## Introduction

Alcohol ethoxylates (AEs) with a general formula of CH_3_(CH_2_)_n_(OCH_2_CH_2_)_y_OH (n: 11–15, 17 and y: 0–18) are one of the most important classes of nonionic surfactants. They are widely used in industrial and commercial formulations as detergents, emulsion stabilizers, paints, dispersing agents, pesticides, petroleum recovery chemicals, foaming agents and wetting enhancers^1–4^. The widespread use of these chemicals has led to a growing concern about their impact on the environment. The primary route of disposal of AEs is down the drain, through sewage systems, and into municipal wastewater treatment plants and as a result, the concentration of AEs in wastewater may reach high levels depending on their consumptions^5–8^. Morrall et al. reported that the concentration of AEs could vary in a wide range from 35.4 to 2717 μg/L in different stages of municipal wastewater treatment plants in the USA^5,9^. While AEs are considered as biodegradable surfactants, in particular, their metabolites such as polyethylene glycol and carboxylated AE chains^10,11^ have the potential to act as endocrine disrupting agents in aquatic organisms, wildlife and even humans^1,12^. Therefore, the degree of treatment of AEs is a key parameter for accurately assessing the potential risk to the environment posed by their release^8,9,13–15^. Until now, advanced oxidation processes (AOPs) based on either HO^·−^^4,16^ or SO_4_^·−^^1,2,4^ radical production have been successfully applied for the treatment of AEs. Published data demonstrated that AEs could be effectively removed from aqueous solutions when some operating parameters such as initial pH, the applied oxidant dose and reaction time were selected properly. On the other hand, there are still some critical issues such as presence anions (Cl^−^, ${\text{HCO}}_{3}^{-}\text{/}{\text{CO}}_{3}^{2-}\text{)}$ in their application to real water/wastewater matrix that need to be further addressed. These anions may serve as radical scavengers and quench HO^·−^ and SO_4_^·−^ to produce less reactive or more selective radicals^17–20^.

In SO_4_^·−^ based AOPs, persulfate directly reacts with organic pollutants to form SO_4_^·−^ radicals that propagate secondary reactions or may create organic or inorganic radicals^21^. If chloride is present in the water/wastewater matrix or originated from organic pollutant itself, it scavenges SO_4_^·−^ to produce less reactive chlorine radicals (Cl^·^, Cl_2_^·^, and ClOH^·−^) or directly reduces persulfate (PS) to generate free chlorine species (Cl_2_, HOCl and OCl^−^)^17,18^. Depending on chemical structure of model target to be degraded, reaction solution pH, and water matrix constituents, some of these chlorine radicals can become dominant species which react with organic molecules through H-abstraction, one-electron oxidation and addition to unsaturated C−C bonds^22,23^. Cl^·^ which is a primary product (Rxn (4)), initiates a cascade of reactions are driven by secondarily formed reactive species (Table 1)^17,18,24^. It reacts with Cl^−^, ^·^OH or H_2_O to form Cl_2_^·−^ or ClOH^·−^^17,22,24,25^. Under the certain reaction conditions, Cl_2_^·−^ can become the predominant radical. Therefore, SO_4_^·−^ based AOPs involved chloride^17,25–27^ have attracted scientific interest, particularly in the saline wastewater treatment^17^.

**Table 1:** Possible reactions of persulfate with chloride ions.

Reaction	Rate constant	Reaction no.	References
S_2_O_8_^2−^ + *hv* → 2SO_4_^·−^		(1)	
SO_4_^·−^ + SO_4_^·−^ → S_2_O_8_^2−^	4.0 × 10^8^ M^−1^ s^−1^	(2)	^25,28^
SO_4_^·−^ + S_2_O_8_^2−^ → SO_4_^2−^ + S_2_O_8_^·−^	6.1 × 10^5^ M^−1^ s^−1^	(3)	^18,25^
SO_4_^·−^ + Cl^−^ ⇄ SO_4_^2−^ + Cl^·^	4.7 × 10^8^ M^−1^ s^−1^	(4)	^18,20,25^
SO_4_^2−^ + Cl^·^ → SO_4_^·−^ + Cl^−^	2.5 × 10^8^ M^−1^ s^−1^	(5)	^18,20,25^
Cl^−^ + Cl^·^ → Cl_2_^·−^	6.5 × 10^9^ M^−1^ s^−1^	(6)	^22,25^
Cl_2_^·−^ → Cl^·^ + Cl^−^	1.1 × 10^5^ s^−1^	(7)	^22,25^
Cl_2_^·−^ + Cl_2_^·−^ → Cl_2_ + 2Cl^−^	8.3 × 10^8^ M^−1^ s^−1^	(8)	^22,25^
ClOH^·−^ ⇄ Cl^−^ + ^·^OH	6.1 × 10^9^ s^−1^	(9)	^22,25^
Cl^−^ + ^·^OH → ClOH^·−^	4.3 × 10^9^ M^−1^ s^−1^	(10)	^22,25^
ClOH^·−^ + H^+^ ⇄ Cl^·^ + H_2_O	2.1 × 10^10^ M^−1^ s^−1^	(11)	^22,25^
Cl^·^ + H_2_O → ClOH^·−^ + H^+^	1.6 × 10^5^ M^−1^ s^−1^	(12)	^22,25^
SO_4_^·−^ + OH^−^ → ^·^OH + SO_4_^2−^	6.5 × 10^7^ M^−1^ s^−1^	(13)	^25,28^
SO_4_^·−^ + H_2_O → HSO_4_^−^ + ^·^OH	5.0 × 10^2^ M^−1^ s^−1^	(14)	^25,28^
Cl_2_^·−^ + H_2_O → ClOH^·−^ + H^+^ + Cl^−^	1.3 × 10^3^ M^−1^ s^−1^	(15)	^22,25^

According to Yuan et al.’s data^17^ the amount of Cl^·^ increased when Cl^−^ concentration ranged from 0 to 0.2 mM and reduced with increasing Cl^−^ content due to formation of Cl_2_^·−^ or ClOH^·−^. More than 90% Acid Orange 7 (AO7) was degraded by Cl_2_^·−^ instead of SO_4_^·−^ at Cl^−^  > 10 mM in the pH range from 1 to 7 in their PS/UV experiment performed at the reaction conditions: (K_2_S_2_O_8_)_0_ = 1 mM, initial pH ((pH)_0_) = 6.5, t = 20 min, and [AO7]_0_ = 0.1 mM. In a study^29^, chloride ions exhibited different behaviors on the degradation of p-nitrosodimethylaniline (RNO) using as a model target depending on the persulfate activation method applied. In the case of heat activation at 65 °C, presence of chloride at a low concentration of 1 mM accelerated degradation rate of RNO. The bleaching rate of RNO increased with increasing chloride concentration (1–400 mM) in the case of alkaline activation at pH 12.4, while the effect of chloride (5–50 mM) on the bleaching rate of RNO found to be insignificant when iron activation (80 mM Fe^2+^) was applied at neutral pH. The accelerating (catalytic) effect of chloride ions on pollutant degradation rate was also reported by Anipsitakis et al.^26^ for phenol and 2,4-dichlorophenol degradations by cobalt activated peroxymonosulfate oxidation, and Fan et al.^30^ for the degradation of sulfamethazine by heat-activated persulfate oxidation process. Some studies have reported a threshold concentration at which the negative effect of chloride begins to be observed^17,25,31^. This threshold chloride concentration was determined as 100 mM for the degradation of Acid Orange 7 (0.1 mM) by PS/UV ([K_2_S_2_O_8_]_0_ = 2 mM; t = 180 min)^17^ and 200 mM for the thermally activated persulfate oxidation of trichloroethylene at 20 °C^25^. The chloride ions had also a negative effect on the degradation of some organic pollutants by SO_4_^·−^ based AOPs^23,25,27,32–35^. Yang et al.^32^ showed the delaying effect of high concentration of Cl^−^ (0.10 and 0.50 M) on the decolorization of an azo dye (Orange7) by microwave-activated persulfate oxidation. The degradation of polyvinyl alcohol (PVA) by UV/S_2_O_8_^2−^ ^36^ and Orange G by the persulfate/Fe^2+^ reagent adversely affected by addition of 100 mM Cl^−^^37^. Gu et al. ^34^ reported both and HCO_3_^−^ and Cl^−^ (1 and 100 mM) had significant scavenging effects on 1,1,1-trichloroethane (TCA) removal by UV/S_2_O_8_^2−^.

Considering the above-mentioned dual effect (inhibitory or accelerating) of chloride ions on the degradation efficiency of SO_4_^·−^ based AOPs, the present study aimed at determining the effect of chloride ions on the removal of an alcohol ethoxylate by PS/UV-C oxidation. The effect of chloride ions on the process performance at an extremely acidic pH of 3.0 and a slightly neutral pH of 6.0 was comparatively examined at a wide range of chloride concentration (50–1000 mg/L) in terms of removals of model target and total organic carbon as well as PS consumption. Further experimental study was performed to determine the effect of organic or inorganic components present in the effluent of a domestic wastewater treatment plant on Brij 30 removal by PS/UV-C oxidation. For this purpose, PS/UV-C oxidations were performed using Brij 30 aqueous solution as well as a domestic wastewater treatment plant effluent spiked with Brij 30 to represent the real wastewater matrix. It should be emphasized that, to the authors’ knowledge, there is no study in the literature investigating the effect of pH-dependent chloride ions on the PS/UV-C oxidation efficiency.

## Materials and methods

### Model alcohol ethoxylate and other chemicals

**Figure 1:**
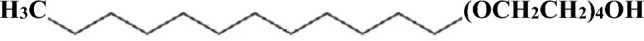
Chemical structure of poly(oxyethylene) (4) lauryl ether (Brij 30).

Pure poly(oxyethylene) (4) lauryl ether [C_12_H_25_(OCH_2_CH_2_)_4_OH], trade name Brij 30, was obtained from Sigma Aldrich (USA). In the structure of the model AE, the number of carbons in the alkyl chain is 12 (C12) and the number of ethoxylates added at the end of the alkyl chain is 4 (E4) as shown in Fig. 1.

Potassium persulfate (K_2_S_2_O_8_) and hydrogen peroxide (H_2_O_2_) used as oxidants were purchased from Sigma-Aldrich (USA). NaCl for chloride addition and H_2_SO_4_ and NaOH for pH adjustment were supplied from MERCK Millipore (Germany). All chemicals used in the experimental study were of analytical grade.

### Samples

Experimental study was performed using both synthetically prepared samples and an effluent from a domestic wastewater treatment plant (WWTP). Synthetic samples were prepared by dissolving a required amount of Brij 30 in distilled water. The WWTP effluent was taken from the final filtration and UV disinfection effluent of an advanced biological treatment plant operating with nutrient and carbon removal. The characteristics of the WWTP effluent are presented in Table 2.

**Table 2:** The character of the WWTP effluent.

	Unit	Concentration
pH	–	8.1
TOC	mg/L	10
DOC	mg/L	9
COD	mg/L	< 30
Chloride	mg Cl/L	110
SO_4_	mg/L	100
Alkalinity	mg CaCO_3_/L	137
Total hardness	mg CaCO_3_/L	476

### Photo-reactor and UV source

**Figure 2:**
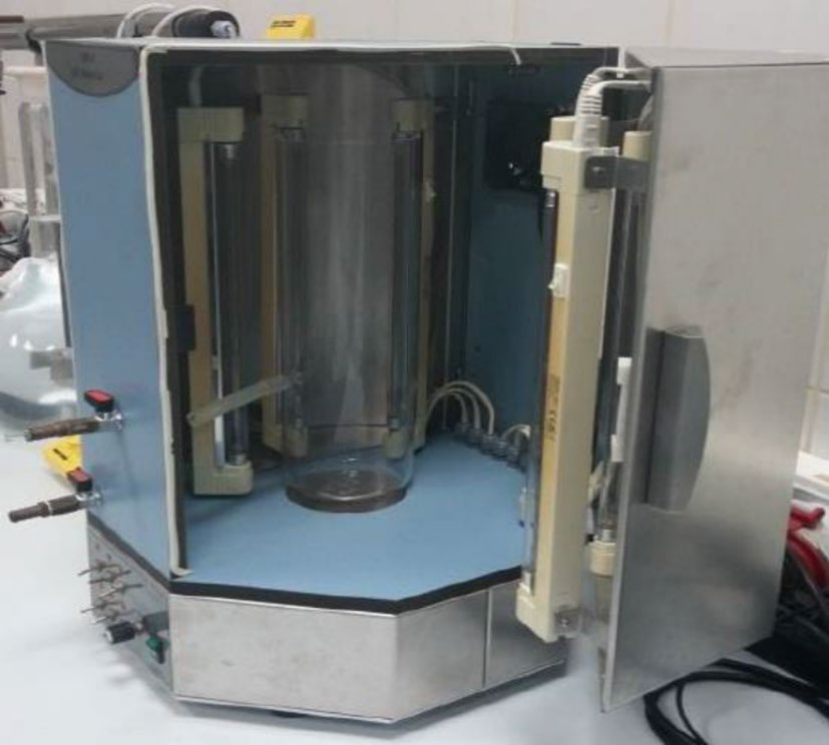
Cylindrical quartz reactor and photo-oxidation chamber.

The cylindrical photo-reactor had a height of 30.0 cm, a diameter of 20.0 cm, and a solution capacity of 2000 mL. Quartz was chosen as the reactor material to allow for easy penetration of UV emissions into all the chemicals present in the reaction solution, thereby maximizing reactive radical production. The chamber was equipped with six 8.0 W UV-C (254 nm) lamps (25 W/m^2^) to provide UV radiation. The spectral distribution of the UV-C lamps had a Gaussian shape with a central wavelength of 254 nm. The metal chamber was ventilated using an air-cooling fan to regulate the internal temperature (25 ± 2 °C). The reactor contents were mixed with a magnetic stirrer at a constant speed of 100 rpm during the reaction. The sampling outlet, which was positioned 12.0 cm above the lowest point of the quartz reactor, was connected to the valve inlet in the chamber via a hose. Samples were collected from the completely mixed reactor contents through the sampling valve located outside the chamber without opening the chamber and stopping the reaction. The photo-reactor setup was shown in Fig. 2^31^.

### Analytical procedures and instruments used in analyses

The concentration of Brij 30 was measured using a modified version^38^ of The Spectrometric Iodine/Iodide (I–I) method defined by Baleux and Champetier^39^. The colorimetric method was used to measure persulfate in solution, as described in more detail elsewhere^1^. Total organic carbon (TOC) measurements were performed to determine the level of mineralization reached during photocatalytic oxidation. SHIMADZU V_CPN_ TOC analyzer performing catalytic oxidative combustion at 680 °C, with auto sampler, using an infrared detector was used for this purpose. pH measurements were made with a Thermo Scientific Orion 720A + pH Meter. Brij 30 and residual PS measurements were made using Pharmacia LKB-Novaspek II model spectrophotometer. All analytical measurements were performed in triplicate and the average of the closest two was reported.

### Operation conditions

Our previous study^1^ extensively discussed the effect of initial PS concentration, initial pH, and model target concentration on Brij 30 removal by PS/UV-C oxidation. The results showed that Brij 30 was rapidly oxidized within 30 min and there was a significant improvement in mineralization with increasing initial PS concentrations up to 3.0 mM at initial pH values of 3.0 and 6.0. Based on these results, operation conditions were selected as [PS]_0_ = 2.3 mM (= 621,8 mg/L), pH_0_ = 3 and 6 for 20 mg/L BAS in the present study. In the case of WWTP effluent, these operating conditions were slightly changed to determine the effect of organic or inorganic components present in the WWTP effluent on Brij 30 removal by PS/UV-C. The effluent was subjected to PS/UV-C treatment in the first set experiments by adding the amount of oxidant required to oxidize all of the organic matter in the sample. This sample was referred to as “Control” sample. The Control sample was subjected to PS/UV-C oxidation at two different pH values: pH 2.8 and the original pH (8.1) of the sample. The purpose of reducing the pH of the sample to 2.8 was to eliminate the alkalinity components (${\text{HCO}}_{3}^{-}\text{/}{\text{CO}}_{3}^{2-}$) by purging as CO_2_. This resulted in a sample (Control I) that was used to study the effect of chloride ions alone on process efficiency. In the third set experiments, the WWTP effluent was used as is without any processing. To compare the process performance of PS/UV-C with that of H_2_O_2_, oxidation experiments were repeated on the WWTP effluent with and without organic matter using H_2_O_2_ as an oxidant under the same reaction conditions.

### Procedure

Prior to commencing PS/UV-C oxidation, the UV-C lamps in the photo-oxidation chambers were turned on for 30 min to stabilize irradiation. After adjusting the pH of the samples with or without NaCl using NaOH and/or H_2_SO_4_ solutions, if necessary, the desired amount of oxidant was added under complete mixing conditions in the dark. The sample was then immediately transferred to the quartz reactor and placed in the chamber. Samples were withdrawn at regular time intervals, and process performance was monitored by measuring Brij 30, TOC, and residual PS.

### Kinetics

The removal of pollutants can be described using pseudo first-order kinetics, as shown below:

16 $$ \frac{{{\text{dC}}}}{{{\text{dt}}}} = - {\text{k}} \times {\text{C}} $$

C represents the pollutant concentration at time t, and k is the pseudo first-order reaction rate constant. Integrating Eq. (16) results in Eq. (17), which is as follows:

17 $${\text{ln}}\frac{\text{C}}{{\text{C}}_{0}}= \text{ } - {k}_{obs}\times \text{t}$$

C_0_ represents the initial pollutant concentration, and *k*_*obs*_ is the observed degradation rate constant.

## Results and discussions

### Aqueous Brij 30 solution

**Figure 3:**
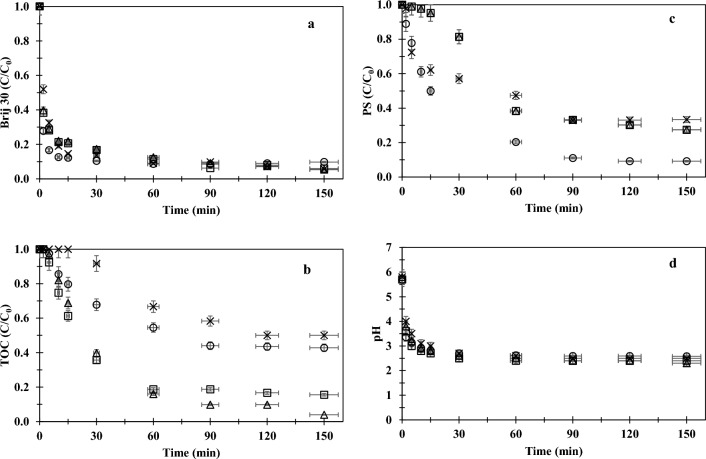
Effect of chloride ions on PS/UV-C oxidation performance ((Brij 30)_0_ = 20 mg/L, (TOC)_0_ = 13 mg/L, pH_0_ = 6.0, [PS]_0_ = 2.3 mM; Cl^−^: 0 (○), 50 mg/L (Δ), 100 mg/L (□), 1000 mg/L ( ×)).

The effect of chloride ions on the PS/UV-C oxidation performance was examined at a wide range of chloride concentrations (50–1000 mg/L) using a reasonably low initial PS concentration of 2.3 mM, at the original pH of the sample (6.0) for a 20 mg/L BAS. Experimental data obtained from this set were illustrated in Figs. 3 (a-d).

Although a reduction in the degradation rate of Brij 30 was observed (Fig. 3a) in the presence of chloride ions, particularly at a chloride concentration of 1000 mg/L, the difference in the degradation rates disappeared after 30 min. 90% Brij 30 degraded in the first 60 min of oxidation for all chloride concentrations tested (Fig. 3a). The degradation of Brij 30 occurred rapidly, accompanied by a minor change in the TOC concentrations (Fig. 3b) during the initial phase of oxidation. This degradation was ascribed to the direct attack of radicals on the ethoxy units of the surfactant^1,31^. As can be seen from Fig. 3d, the solution pH sharply dropped from 6.0 to 3.0–4.0 within 5 min in all oxidation applications. The drop in the solution pH could be attributed to the presence of oxidation products such as formates with shorter ethoxy chains, aldehydes and smaller ethoxy in the reaction solution ^1,31^. The formation of formic acid was also supported by the relatively constant pH (Fig. 3d) which prevailed after the rapid oxidation phase due to its strong buffer effect^1^. Gu et al.^34^ also reported a drop in pH from 5.6 to 3.1 during UV/S_2_O_8_^2−^ oxidation of TCA, which was explained by the production of protons and acid by-products.

The analysis of the data demonstrated that the removal of TOC followed the pseudo first-order kinetics (Eq. 16). A comparison of rate constants (*k*_*obs*_) given in Table 3 indicated that the fastest organic matter removal was achieved at 50 mg/L chloride concentration. The acceleration in mineralization at this concentration was likely due to reactions involving reactive chlorine species, which reduced the sink of SO_4_^·−^ by Cl^−^ scavenging^30^. A decrease in the recombination frequency of SO_4_^·−^ (Eq. 2) through chain reactions (given in Table 1) could be shown as another mechanism responsible for accelerating mineralization^17^. A slight slowdown in mineralization was observed, as the chloride concentration increased from 50 to 100 mg/L, resulting in a marked reduction in organic matter removal efficiency. While almost complete mineralization (96% TOC removal) occurred at 50 mg/L chloride, organic matter removal efficiency reduced to 84% at 100 mg/L chloride at the end of the PS/UV-C oxidation. When the chloride concentration was increased to 1000 mg/L, the retarding effect of chloride ions was observed and no change in TOC concentration or PS concentration was detected during the first 15 min of the reaction. After a lag period of 30 min, mineralization started to occur in parallel with the PS consumption, resulting in only 50% removal of TOC within 120 min. No further TOC removal observed thereafter. This significant reduction in organic matter removal at a relatively high chloride concentration of 1000 mg/L was attributed to the conversion of a greater proportion of to SO_4_^·−^ less reactive chlorinated radicals^17^. The presence of high levels of chloride ions probably promoted the formation of these radicals, which were more selective than SO_4_^·−^ radicals in oxidizing organic matter^40–42^.

**Table 3:** The pseudo first order reaction rates (R^2^ ≥ 0.99; t = 0–90 min).

Chloride (mg/L)	*k*_*obs,TOC*_ (1/min)	TOC removal^a^ (%)
Initial pH: 6		
0	0.0081	57
50	0.032	96
100	0.0281	84
1000	0.0058^b^	50
Initial pH: 3		
0	0.0357	92
30	0.0676	92
750	0.0173	78

^a^overall TOC removal; ^b^for time period of 15–90 min.

**Figure 4:**
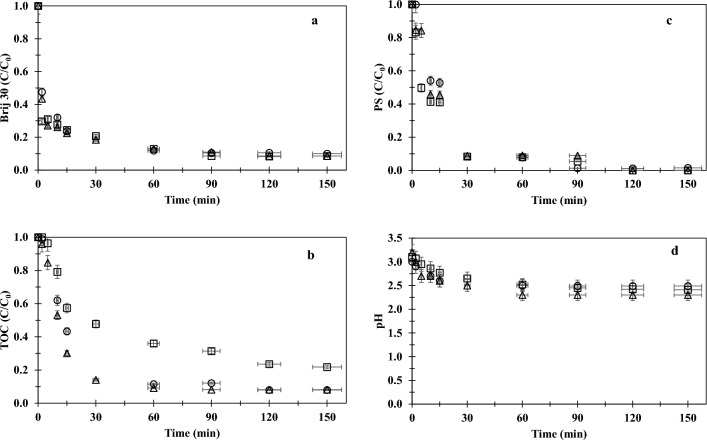
Effect of chloride ions on PS/UV-C oxidation performance ((Brij 30)_0_ = 20 mg/L, (TOC)_0_ = 13 mg/L, pH_0_ = 3.0, [PS]_0_ = 2.3 mM; Cl^−^: 0 (○), 30 mg/L (Δ), 750 mg/L (□)).

In order to determine the effect of initial pH on PS/UV-C oxidation efficiency, a further experimental study was conducted at pH 3 in parallel with the initial set. The data obtained from this study are presented in Fig. 4.

Upon comparing Figs. 3a,b and 4a, b, it can be concluded that both Brij 30 degradation and mineralization (TOC abatement) achieved through PS/UV-C oxidation at an initial pH of 3.0 followed a similar pattern to that of the PS/UV-C oxidations initiated at pH 6.0. Similar conclusions can be drawn for the PS/UV-C oxidation of the Brij 30 model target in the acidic medium (pH 3), except for the PS consumption (Fig. 4c) during the course and the initial pH drop that had already occurred upon the addition of acid. When PS/UV-C oxidation was initiated at pH 3.0, the PS consumption was relatively rapid, resulting in faster mineralization than that of initial pH 6.0 (Table 3). After 120 min, the PS was completely consumed in all experiments.

The influence of the initial pH was more pronounced on the TOC removal rates. The rate constants (*k*_*obs,TOK*_) of the initial pH of 3 were twofold higher than those of the initial pH of 6 (Table 3), indicating faster mineralization in 90 min of PS/UV-C oxidation. This increase in mineralization rate can be explained by the formation of more sulfate radicals due to the catalyzing effect of the acid (Eqs. 18 and 19) as reported in the literature^1,23,34,36,37,43,44^.

18 $$ {\text{S}}_{{2}} {\text{O}}_{{8}}^{{{2} - }} + {\text{ H}}^{ + } \to {\text{ HS}}_{{2}} {\text{O}}_{{8}}^{ - } $$

19 $$ {\text{HS}}_{{2}} {\text{O}}_{{8}}^{ - } \to {\text{ SO}}_{{4}}^{\cdot - } + {\text{ SO}}_{{4}}^{{{2} - }} + {\text{ H}}^{ + } $$

Furthermore, the extremely acidic conditions suppressed the adverse effect of excess chloride ions. This deduction can be also supported by the time-based TOC data acquired for the 750 mg/L chloride sample. During the PS/UV-C oxidation process, the degradation of organic matter (TOC) commenced at the 5th minute and culminated in a 78% reduction by the end of the treatment. In the presence of excess chloride ions, the initiation of oxidation under acidic conditions (*k*_*obs,TOC*_ = 0.0173 min^−1^; 750 mg Cl^−^/L) resulted in an additional 22% TOC removal compared to the initiation at pH 6 (*k*_*obs,TOC*_ = 0.0058 min^−1^; 1000 mg Cl^−^/L). Based on the TOC data, it can be noted that initiating PS/UV-C photocatalytic oxidation under acidic pH conditions in chloride-containing samples would not only enhance the removal efficiency of organic matter, but would also cause this process to proceed more rapidly. However, it is important to note that acidic conditions may favor the formation of chlorate (${{\text{C}}{\text{l}}{\text{O}}}_{3}^{-}$), classified as harmful to health and the ecosystem^24^.

### Wastewater treatment plant effluent

**Figure 5:**
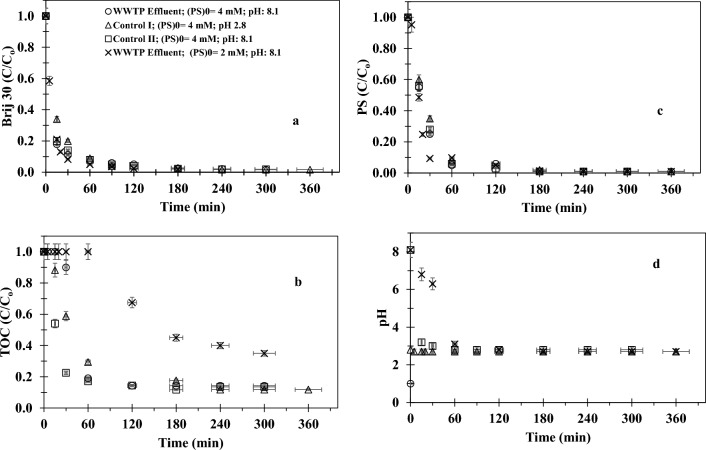
Results of PS/UV-C oxidation of the WWTP effluent.

Figure 5 illustrates the results of PS/UV-C oxidation of the WWTP effluent. The WWTP effluent spiked with 20 mg/L Brij 30 was first subjected to PS/UV-C oxidation at an initial PS concentration of 2 mM (= 540,7 mg/L) and its original pH (8.1) in parallel with the oxidation experiments carried out on the BAS sample. Although, Brij 30 was completely degraded in the first 90 min of this PS/UV-C oxidation, no change in the TOC content was observed in the first 60 min. The TOC concentration began to reduce when the solution pH gradually decreased from 8.1 to 3.9 after 60 min. Limited TOC removal (65%) was achieved in this experiment, likely due to insufficient oxidant. As the WWTP effluent contains additional organic matter (10 mg TOC/L) equivalent to Brij 30 (13 mg TOC/L), the oxidant requirement increased to degrade the organic matter corresponding to 23 mg/L TOC equivalent in the effluent^16^. Consequently, the oxidant concentration was increased to 4 mM. Increasing the PS concentration from 2 to 4 mM (1081 mg/L) reduced the lag period for organic matter degradation from 60 to 30 min. Mineralization increased significantly as the pH of the solution decreased to 3.1, reaching 81% within 60 min. There was no change in residual TOC concentration (2.3 mg/L) at longer reaction times as the PS was fully consumed. Based on the TOC data, the initial PS dose was set at 4 mM for further experiments aimed at assessing the impact of organic and inorganic content in the WWTP effluent on oxidation efficiency.

Analyzing the data presented in Fig. 5a,c indicated that the presence of other inorganic and organic content in the WWTP effluent had a negligible effect on both the model target Brij 30 removal and PS consumption. On the other hand, TOC removal was significantly influenced by the WWTP effluent matrix, specifically at the earlier stage of oxidation. Figures 5a,b,d demonstrated that TOC removal began once acidic pH levels were reached, suggesting that Brij 30 was degraded to intermediates through proton-producing reactions. As seen from Table 4, TOC removal efficiency varied depending on the organic and inorganic content present in the WWTP effluent in the initial 15 and 30 min. The fastest TOC removal was obtained in the first 30 min of PS/UV-C oxidation performed with the Control II sample bearing 110 mg/L Cl^−^ and 164 mg/L ${\text{HCO}}_{3}^{-}$. In the Control I sample, which contained only chloride ions, TOC removal was initially slower compared to the Control II sample. A comparison of the TOC data of Controls I and II revealed a positive effect of ${\text{HCO}}_{3}^{-}/{\text{CO}}_{3}^{{2}-}$ anions, as previously documented^45–47^. This effect was explained by formation of CO_3_^·−^ radicals^42^.

20 $$ {\text{SO}}_{{4}}^{\cdot - } + {\text{HCO}}_{{3}}^{ - } \to {\text{SO}}_{4}^{{{2} - }} + {\text{HCO}}_{{3}}^{\cdot - } \to \left( {k = 2.8 - 9.1 \times 10^{6} {\text{M}}^{{ - {1}}} {\text{s}}^{{ - {1}}} } \right) $$

21 $$ {\text{SO}}_{{4}}^{\cdot - } + {\text{CO}}_{{3}}^{2 - } \to {\text{SO}}_{4}^{{{2} - }} + {\text{CO}}_{{3}}^{\cdot - } \to \left( {k = 6.1 - 410 \times 10^{6} {\text{M}}^{{ - {1}}} {\text{s}}^{{ - {1}}} } \right) $$

**Table 4:** The effect of organic and inorganic components present in the WWTP effluent on PS/UV-C and H_2_O_2_/UV-C performances ([PS]_0_ or [H_2_O_2_]_0_ = 4 mM).

	Initial pH	Organic Matter^a^	Cl^−^	Alkalinity species	15 min		30 min		120 min	
					TOC^b^	pH^c^	TOC^b^	pH^1^	TOC^2^	pH^3^
PS/UV oxidation										
The WWTP effluent	8.1	+	+	**+ **	−	6.8	14%	6.3	86%	2.8
Control I	2.8	−	+	−	12%	2.6	41%	2.7	82%	2.7
Control II	8.1	−	+	+	46%	3.2	77%	3.0	86%	2.8
H_2_O_2_/UV oxidation										
The WWTP effluent	8.1	+	+	+	−	6.8	30%	6.7	77%	6.6
Control II	8.1	−	+	+	−	6.6	30%	6.2	62%	6.5

^1^organic matter in the WWTP effluent itself; ^2^removal efficiency (%); ^3^pH measured at the specified minute of the oxidation; (+) present or (−) absent in the sample content.

HCO_3_^·−^/CO_3_^·−^ which are moderately reactive radicals, can react preferentially with electron rich compounds^45–47^. Despite the fact that the presence of bicarbonate promoted the removal of TOC, its participation in the reaction needs to be further studied, since the pH of the Control II sample dropped to 3.9 after 15 min of PS/UV-C oxidation and all the bicarbonate was converted to carbon dioxide at this pH.

When WWTP effluent was used as is, a lag period in TOC removal was observed at the beginning of PS/UV-C oxidation, most likely due to its structure of initial organic matter content of the effluent, and a limited TOC removal of 14% was attained in 30 min. However, the negative effects were eventually suppressed, and at the end of all PS/UV-C oxidations, almost equal TOC removal efficiencies were achieved. A lag period in TOC removal was also observed for H_2_O_2_/UV-C oxidation, resulting in lower TOC removal efficiencies than for PS/UV-C. The adverse effect of organic matters present in water matrix on the SO_4_^·−^ and HO^·−^ based AOPs has been also mentioned in the relevant literature^24,30,34,45–47^. Fan et al.^30^ reported that the presence of fulvic acid (SRFA) within the range of 0–10 mg/L had a negative effect on the degradation of sulfamethazine (SMZ) by heat-activated PS oxidation and presence of ${\text{HCO}}_{3}^{-}$ and Cl^−^ ions (0–50 mM) exhibited a promoting effect on the process performance. Gu et al.^34^ observed a lag phase of the UV/S_2_O_8_^2−^ oxidation of TCA when humic acid was present at a concentration of 10 mg/L. Similarly, Xu et al.^47^ confirmed the inhibitory effect of natural organic matters (NOMs) on the degradation of tetracycline during the UVC/persulfate process. This inhibitory effect was speculatively attributed to the light competition or quenching of reactive oxygen species by organic matter present in the WWTP effluent itself ^46,47^.

## Conclusions

The present study proved that the model pollutant Brij 30 was effectively degraded by SO_4_^·−^ based oxidation under favorable reaction conditions. However, the mineralization rate of its intermediates was strongly dependent on the water matrix. In the case of BAS, chloride ions had a role in accelerating the mineralization of intermediates. This accelerating effect of chloride ions was more pronounced, when the PS/UV-C process commenced upon acidic conditions promoting the formation of more sulfate radicals due to acid-catalyzing effect.

The TOC data revealed incomplete mineralization, corresponding to an 86% TOC removal upon degradation of Brij 30 in combination with inert organic matter from the WWTP effluent. The findings indicated that organic and inorganic compounds present in the wastewater matrix played a significant role in the degradation of Brij 30 model target intermediates by chloride-containing SO_4_^·−^ based AOPs. The impact of these components on the performance of PS/UV-C oxidation was ranked in the following order of increasing positive effects: (Organic matter + Cl^−^ +${\text{HCO}}_{3}^{-}$) < (Cl^−^) < (Cl^−^ +${\text{HCO}}_{3}^{-}$). While presence of organic matter together with Cl^−^ and ${\text{HCO}}_{3}^{-}$ anions with yielded the slowest oxidation, co-existence of ${\text{HCO}}_{3}^{-}$ and Cl^−^ anions promoted the mineralization of Brij 30, resulted in the fastest removal of organic matter at the early stage of the PS/UV-C oxidation. This promoting effect diminished in the presence of chloride alone. It is worth noting that the replacement of the PS/UV-C process with H_2_O_2_/UV-C oxidation alleviated the adverse effect of organic matter on the process rate. However, considering that lower TOC removal efficiencies were obtained with the H_2_O_2_/UV-C oxidation compared to the PS/UV-C oxidation in all cases tested in this study, it can be said that the H_2_O_2_/UV-C process exhibited a more sensitive structure to the wastewater matrix.

## Data Availability

All data supporting the findings of this research are included in the paper.
